# The prognostic value of negative lymph node count for patients with gastric cancer who received preoperative radiotherapy

**DOI:** 10.18632/oncotarget.14943

**Published:** 2017-02-01

**Authors:** Xinxing Li, Weigang Zhang, Xianwen Zhang, Haolu Wang, Kai Xu, Houshan Yao, Jun Yao, Xiaowen Liang, Zhiqian Hu

**Affiliations:** ^1^ Department of General Surgery, Changzheng Hospital, The Second Military Medical University, Shanghai 200003, China; ^2^ Therapeutics Research Centre, School of Medicine, The University of Queensland, Princess Alexandra Hospital, Woolloongabba, QLD 4102, Australia

**Keywords:** gastric cancer, preoperative radiotherapy, negative lymph node, survival

## Abstract

Negative lymph node (NLN) count provides accurate prognostic information in patients with gastric cancer. However, it is unclear whether NLN still has prognostic value for patients received preoperative radiotherapy. In this study, Surveillance, Epidemiology, and End Results Program (SEER)-registered gastric cancer patients were used for analysis. Clinicopathological characteristics and survival time were collected. Univariate and multivariate Cox proportional hazards models were used to assess the risk factors for survival. NLN count was validated as an independent prognostic factor in both univariate and mulivariate analysis (P < 0.001). X-tile plots identified 12 as the optimal cutoff value to divide the patients into high and low risk subsets in terms of survival rate. Nomogram based on cancer-specific survival was successfully established according to all significant factors. The C-index was 0.630 (95% CI: 0.605–0.655). Subgroup analysis showed that NLN count was a prognosis factor for patients with advanced gastric cancer (stage ypII and ypIII). In conclusion, our results firmly demonstrated that NLN count was an independent prognostic factor for patients with gastric cancer who received preoperative radiotherapy. It provides more accurate prognostic information especially for patients with advanced gastric cancer (stage ypII and ypIII). Nomograms based on cancer-specific survival could be recommended as practical models to evaluate prognosis.

## INTRODUCTION

Gastric cancer (GC) is the fourth most common malignancy and the second leading cause of cancer related death worldwide [1]. Surgical resection is the only curative option, but outcomes are poor [2]. Preoperative radiotherapy (Pre-RT) offers multiple advantages. Trials from Europe and Asia have been conducted to determine the feasibility of Pre-RT for GC [3]. Several randomized studies and meta-analysis have demonstrated a survival benefit for Pre-RT in patients with GC compared with surgery alone [3–5].

Lymph node (LN) metastases indicate worse treatment response and poorer survival. The American Joint Committee on Cancer (AJCC) successively established standards for LN stage based on metastatic LNs in the anatomical positions and the number of LN metastasis [2]. While the node-positive patients with GC are heterogeneous and the prognosis of these patients cannot be stratified by the node-stage only [6, 7]. Therefore, the concept of negative lymph node (NLN) counts attracted attention recently. It can serve as a prognostic indicator in various cancers, such as cervical [8], breast [9], esophagus [10] and GC [11]. However, Pre-RT can yield tumor downstaging, reduce the burden of residual microscopic disease at surgery and reduce the number of LNs retrieved in operation [3]. With the decreased LNs retrieval, the prognostic value of the LN count might also diminish [12]. Thus, it is unclear whether NLN still has prognostic value for survival of patients with GC who received Pre-RT. The purpose of this study was to assess the association between NLN count and survival of patients with GC who received Pre-RT. In order to get convincing results in a larger series of patients, we used the SEER (Surveillance, Epidemiology and End Results)-registered database to analyze this association, and determine the optimal cutoff value of NLN count.

## RESULTS

### Patient characteristics in SEER database

In our study period from 2004 to 2013, a total of 1,346 patients with GC who received Pre-RT met our selection criteria, including 1,130 male and 216 female. The median age of patients was 62 years (20 - 93). There were 738 patients with stage ypN0, 297 with stage ypN1, 214 with stage ypN2 and 97 with stage ypN3. The demographics and pathological features of patients are summarized in Table 1. The ypN stage was closely correlated with sex, year of diagnosis, grade, histologic type, and ypT stage (P < 0.05).

**Table 1:** Demographics and pathological features of patients with GC who received Pre-RT

Variable	N (1346)	ypN0	ypN1	ypN2	ypN3	χ2	P value
		N (%)	N (%)	N (%)	N (%)		
Sex							
Male	1130	595 (52.7)	267 (23.6)	189 (16.7)	79 (7.0)	17.347	0.001
Female	216	143 (66.2)	30 (13.9)	25 (11.6)	18 (8.3)		
Age							
<60	526	267 (50.8)	121 (23.0)	93 (17.7)	45 (8.6)	6.854	0.077
≥60	820	471 (57.4)	176 (21.5)	121 (14.8)	52 (6.3)		
Year of diagnosis							
2004-2008	495	259 (52.3)	114 (23.0)	73 (14.7)	49 (9.9)	9.756	0.021
2009-2013	851	479 (56.3)	183 (21.5)	141 (16.6)	48 (5.6)		
Race							
White	1202	662 (55.1)	267 (22.2)	186 (15.5)	87 (7.2)	3.846	0.698
Black	59	29 (49.2)	11 (18.6)	13 (22.0)	6 (10.2)		
Others	85	47 (55.3)	19 (22.4)	15 (17.6)	4 (4.7)		
Grade							
Well-moderately differentiated	487	311 (63.9)	110 (22.6)	52 (10.7)	14 (28.7)	71.162	<0.001
Poor-undifferentiated	728	337 (46.3)	165 (22.7)	153 (21.0)	73 (10.1)		
Unkown	131	90 (68.7)	22 (16.8)	9 (6.9)	10 (7.6)		
Histologic type							
Adenocarcinoma	1113	626 (56.2)	245 (22.0)	175 (15.7)	67 (6.0)	23.931	0.001
Signet ring cell carcinoma	159	70 (44.0)	33 (20.8)	33 (20.8)	23 (14.5)		
Others	74	42 (56.8)	19 (25.7)	6 (8.1)	7 (9.5)		
ypT Stage							
T1	136	102 (75.0)	20 (14.7)	12 (8.8)	2 (1.5)	44.418	<0.001
T2	182	110 (60.4)	46 (25.3)	17 (9.3)	9 (4.9)		
T3	576	309 (53.6)	121 (21.0)	95 (16.5)	51 (8.9)		
T4	452	217 (48.0)	110 (24.3)	90 (19.9)	35 (7.7)		

### The optimal cutoff value for NLNs determined by X-tile program

**Figure 1:**
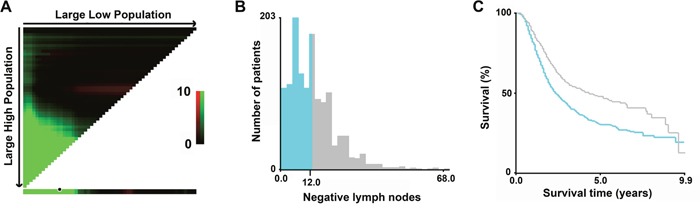
X-tile analysis of survival data from the SEER registry X-tile analysis was performed using patients’ data from the SEER registry, equally divided into training and validation sets. X-tile plots of the training sets are shown with plots of matched validation sets shown in the smaller inset **A**. The optimal cut-point highlighted by the black circle in the left panels is shown on a histogram of the entire cohort **B**., and a Kaplan-Meier plot **C**. P values were determined using the cutoff point defined in the training set and applying it to the validation set. (The optimal cutoff value for NLN count is 12, χ^2^= 26.872, P < 0.001.)

To assess the influence of different NLN count on cancer-specific survival (CSS), we analyzed the individual result using different NLN count ranging from 1 to 24. The 3-year and 5-year CSSs were calculated for patients with N (NLNs number) or more nodes and less than N nodes. As shown in Table 2, NLN count was a prognosis factor for number ranging from 1 to 18. The 5-year CSS rate increased from 14.5% to 46.5%. Next X-tile plots were constructed and the maximum χ2 log-rank value of 26.872was produced (Figure 1, P < 0.001), applying 12 as the optimal cutoff value to divide the cohort into high and low risk subsets in terms of CSS. There was a significant difference in 3-year and 5-year CSS between two subsets (42.3% v.s. 56.2%, and 30.5% v.s. 40.8%, respectively).

**Table 2:** Univariate analysis of the influence of different NLN count on CSS in patients with GC who received Pre-RT

Total NLNs	No.	3-year CCS	5-year CCS	Log rank χ2 test	P value	Total NLNs	No.	3-year CCS	5-year CCS	Log rank χ2 test	P value
<1	27	27.2%	14.5%	12.171	<0.001	<13	738	42.3%	30.5%	27.123	<0.001
≥1	1319	48.8%	38.3%			≥13	608	56.2%	48.1%		
<2	69	24.6%	17.4%	22.697	<0.001	<14	798	43.1%	31.8%	21.641	<0.001
≥2	1277	49.7%	39.0%			≥14	548	56.7%	48.0%		
<3	109	27.5%	17.9%	29.529	<0.001	<15	844	43.4%	32.5%	21.094	<0.001
≥3	1237	50.3%	39.7%			≥15	502	57.6%	48.2%		
<4	167	32.3%	24.5%	23.963	<0.001	<16	888	43.6%	32.5%	23.181	<0.001
≥4	1179	50.8%	39.9%			≥16	458	58.5%	50.0%		
<5	223	37.0%	27.4%	18.418	<0.001	<17	938	44.5%	33.6%	16.224	<0.001
≥5	1123	50.7%	40.0%			≥17	408	58.5%	49.4%		
<6	275	39.0%	29.3%	16.654	<0.001	<18	981	45.7%	35.1%	7.492	0.006
≥6	1071	50.9%	40.2%			≥18	365	56.8%	46.5%		
<7	356	39.5%	29.5%	19.387	<0.001	<19	1027	46.6%	36.1%	3.552	0.059
≥7	990	51.7%	41.0%			≥19	319	55.2%	44.4%		
<8	426	39.9%	29.8%	20.603	<0.001	<20	1061	47.0%	36.2%	3.248	0.071
≥8	920	52.5%	41.9%			≥20	285	54.3%	45.4%		
<9	483	40.0%	28.3%	26.671	<0.001	<21	1094	47.2%	36.5%	2.188	0.139
≥9	863	53.3%	44,1%			≥21	252	54.7%	45.2%		
<10	554	41.2%	29.0%	25.278	<0.001	<22	1130	47.4%	36.5%	2.868	0.090
≥10	792	53.6%	45.0%			≥22	216	54.7%	46.9%		
<11	611	41.7%	30.0%	24.380	<0.001	<23	1152	47.6%	36.9%	1.473	0.225
≥11	735	54.2%	45.4%			≥23	194	53.6%	44.9%		
<12	663	42.6^	31.0%	21.310	<0.001	<24	1162	47.7%	37.0%	1.189	0.276
≥12	683	54.3%	45.4%			≥24	184	53.2%	43.9%		

### Nomograms for predicting survival of patients with GC who received Pre-RT

According to univariate analysis, NLN count (P<0.001), sex (P=0.018), year of diagnosis (P=0.045), grade (P=0.012), histologic type (P<0.001) and ypN stage (P<0.001) were associated with poor survival. In multivariate Cox analysis (Table 3), grade, ypN stage and NLN counts were independently prognostic factors. A higher number of NLN counts showed a favorable effect on survival (HR=1.375, 95% CI: 1.166-1.621, P< 0.001).

**Table 3:** Univariate and multivariate survival analysis for evaluating the influence of NLNs on CSS

Variable	3-year CCS	5-year CCS	Univariate analysis		Multivariate analysis	
			Log rank χ2 test	P value	HR (95%CI)	P value
Sex			5.596	0.018		**0.069**
Male	47.0%	36.3%			**Ref**.	
Female	55.3%	45.5%			**1.253 (0.998~1.575)**	
Age			0.728	0.394		NI
<60	49.9%	38.9%				
≥60	47.3%	37.1%				
Year of diagnosis			4.0	0.045		0.389
2004-2008	45.9%	34.9%			Ref.	
2009-2013	50.5%	39.6%			1.702 (0.908~1.264)	
Race			2.461	0.292		NI
White	47.7%	37.4%				
Black	49.1%	36.2%				
Others	59.0%	45.9%				
Grade			20.972	<0.001		0.012
Well-moderately differentiated	55.7%	43.8%			Ref.	
Poor-undifferentiated	43.9%	31.6%			0.886 (0.635-0.981)	
Unkown	41.0%	31.1%			1.233 (0.938-1.465)	
Histologic type			16.316	<0.001		0.091
Adenocarcinoma	50.8%	40.8%			Ref.	
Signet ring cell carcinoma	34.0%	21.6%			1.003 (0.721~1.395)	
Others	44.4%	30.4%			1.298 (0.896~1.878)	
ypT Stage			3.338	0.342		NI
T1	50.4%	44.8%				
T2	52.1%	35.4%				
T3	49.8%	37.5%				
T4	44.5%	36.7%				
ypN Stage			113.780	<0.001		<0.001
ypN0	61.3%	49.2%			Ref.	
ypN1	37.3%	28.8%			0.366 (0.280~0.480)	
ypN2	30.3%	22.4%			0.625 (0.470~0.833)	
ypN3	20.5%	10.5%			0.714 (0.529~0.963)	
No. of NLNs			27.123	<0.001		<0.001
<13	42.3%	30.5%			Ref.	
≥13	56.2%	48.1%			1.375 (1.166~1.621)	

**Figure 2:**
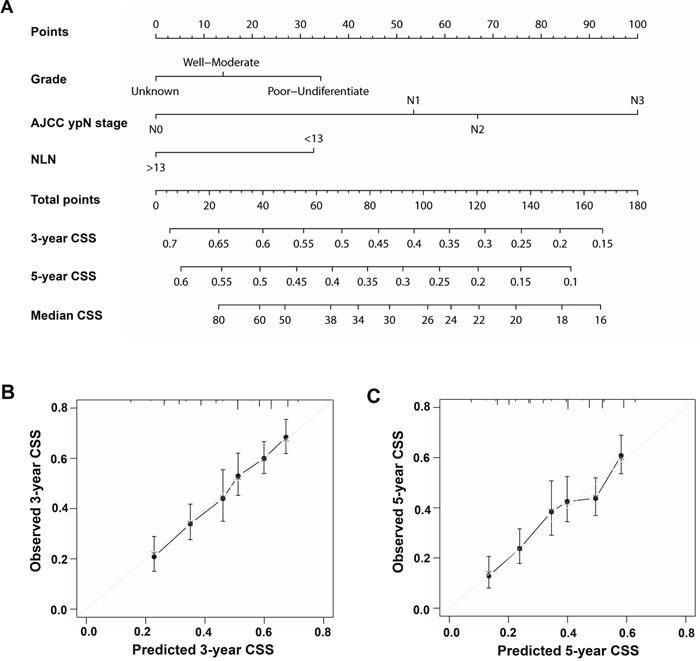
Nomogram for predicting 3-year and 5-year CSS of patients with GC who received Pre-RT **A**. Nomograms with clinicopathological characteristics and NLN count. Nomograms could be interpreted by summing up the points assigned to each variable, which was indicated at the top of scale. The total points could be converted to predicted 3-year and 5-year probability of death for patients with GC who received Pre-RT in the lowest scale. The Harrell's C-index for CSS prediction was 0.630 (95% CI: 0.605–0.655). **B and C**. Calibration curves using nomograms with clinicopathological characteristics and NLNs for predicting 3-year and 5-year CSS. The X-axis was nomogram-predicted CSS and Y-axis was observed CSS. The reference line was 45° and indicated perfect calibration.

In order to predict CSS, the nomogram was established by multivariate Cox regression model according to all significantly independent factors including grade, ypN stage and NLN counts (Figure 2). Nomogram could be interpreted by summing up the points assigned to each variable, which was indicated at the top of scale. The total points could be converted to predicted 3-year and 5-year CSS to obtain the probability of death. The Harrell's C-index for CSS prediction was 0.630 (95% CI: 0.605–0.655). Calibration curves for the nomogram revealed no deviations from the reference line and no need of recalibration (Figure 2).

### Subgroup analysis for evaluating the effect of NLN count according to TNM stage

**Figure 3:**
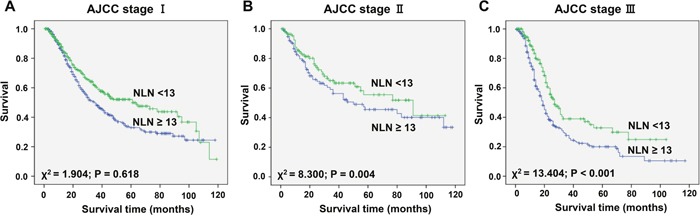
Log-rank tests of CSS comparing patients with NLNs (< 13 VS ≥13) for A. stage ypI: χ^2^ = 1. 904, P = 0.618; B. stage ypII: χ^2^ = 8.300, P = 0.004; and C. stage ypIII: χ^2^ = 13.404, P < 0.001.

According to the AJCC-7 GC staging system, patients with LNs metastases were divided into three subgroups including stage ypI, ypII and ypIII. We then further analyzed the effects of NLN on survival in each subgroup. As shown in Table 4, NLN count was an independently prognostic factor in the stage ypII (χ2= 8.300, P=0.004) and stage ypIII subgroups (χ2= 13.404, P<0.001), but not in the stage ypI subgroup (χ2= 1.904, P=0.168) on both univariate and multivariate analysis (Figure 3, P < 0.001).

**Table 4:** Univariate and multivariate survival analysis for evaluating the influence of the NLN count on CSS in three subgroups

Variable	3-year CCS	5-year CCS	Univariate analysis		Multivariate analysis	
			Log rank χ2 test	P	HR(95%CI)	P
Stage ypI						
No. of NLNs			1.904	0.168	NI	
<13	56.3%	45.5%				
≥13	65.0%	55.5%				
Stage ypII						
No. of NLNs			8.300	0.004		0.019
<13	48.3%	33.0%			Reference	
≥13	60.3%	52.0%			1.316 (1.045~1.656)	
Stage ypIII						
No. of NLNs			13.404	<0.001		0.001
<13	27.7%	20.0%			Reference	
≥13	38.9%	32.8%			1.641 (1.238~2.176)	

## DISCUSSION

Despite a decreasing incidence of GC in some developed countries over the past decade, about 1,000, 000 new cases are diagnosed each year globally, with the 5-year survival less than 30% [13]. LN metastasis is considered one of the most significant prognostic factors [14]. Yet, the number of positive LN is often affected by many facts such as neoadjuvant therapy, and the number of LN retrieved and inspected [3, 15]. Once the LN retrieved is not enough, the prediction of survival would be inaccurate. The intended purpose of Pre-RT is tumor down-staging by decreasing the primary tumor bulk and reducing the burden of residual associated LN metastases at surger [3]. It has been reported that Pre-RT may cause radiation-induced lymphocyte destruction and stromal fibrosis resulting in alterations of the morphology of the LNs, making LN detection during operation more difficult [3]. Some researchers also found that a decreased LN count after Pre-RT was related to good survival [4, 5].

NLN count has a unique advantage that it is little influenced by the number of LN retrieved [11]. The more NLN count is, the better the survival would be. It has been reported that the NLN count was a key factor for the survival of patients with GC after curative resection [16]. In this study, we found that the NLN count was an independent prognosis factor for patients with GC who received Pre-RT. And we also identified the optimal cutoff value for NLN count as 12. Obviously, NLN count is a good supplement for LN stage and TNM stage on evaluating prognosis, especially for patients with advanced GC (stage ypII and ypIII) who received Pre-RT. Until now, there has been no report confirming the mechanism of NLNs influencing on the prognosis of GC. It is suggested that lymphatic micrometastasis is a key etiology of recurrence and metastasis after resection of GC [17]. LN micrometastasis, is common in nodes with the size ranging from 0.2 mm to 2.0 mm which determined to be negative by HE staining, but positive for cytokeratin by immunohistochemicalstaining [18]. It is difficult to find lymphatic micrometastasis during operation. Because the NLN count has potential to reflect the dissection of lymphatic micrometastasis, we can retrieve more NLNs to reduce the residual micrometastases, in order to improve the prognosis of GC. In this study, subgroup analysis showed that NLN count was an independent prognosis factor for GC patients with stage ypII and ypIII, but not for patients with stage ypI. One possible explanation might be that less LN is retrieved in GC patients with stage ypI, thus the prediction of survival would be less accurate.

The results of this study have several potential limitations. First, the SEER database does not include information of therapeutic options such as radical resection or palliative therapy, detailed information of chemotherapy, recurrence and metastasis, which may also impact patients’ prognosis [11]. Especially the preoperative chemotherapy has already been recognized as effective for latent lymph node micrometastasis [19]. It actually may have great impact on the prognosis and NLN count [20]. Thus, additional trials will be needed to investigate whether NLN still has prognostic value count on survival of patients with GC who received chemotherapy. Second, different operative approaches, doctors and even pathologist would affect the detective rate of total LN and metastatic LN, but the SEER do not include these information [12]. Third, preoperative clinical grading and the information about tumor and LN recession response to treatment are still uncertain. All of these factors may influence the curative effect of neoadjuvant therapy and the survival.

In conclusion, our analysis of the SEER database revealed that NLN count (with an optimal cutoff value of 12) in was an independent prognosis factor for patients with GC who received Pre-RT. Subgroup analysis showed that NLN count provided more accurate prognostic information especially for patients with advanced GC (stage ypII and ypIII). Nomograms based on CSS could be recommended as practical models to evaluate prognosis.

## MATERIALS AND METHODS

### Patient selection

All data was obtained from the SEER database. The current SEER database consists of 18 population-based cancer registries that represent approximately 26% of the population in the United States. The SEER data contain no identifiers and are publicly available for studies of cancer-based epidemiology and health policy.

The National Cancer Institute's SEER*Stat software (Surveillance Research Program, National Cancer Institute SEER*Stat software, www.seer.cancer.gov/seerstat (Version 8.3.2), was used to identify patients whose pathological diagnosis as GC between 2004 and 2013. Only patients who underwent preoperative radiotherapy and surgical treatment with age of diagnosis more than 18 years were included. Histological type were limited to adenocarcinoma (8140/3, 8144/3, 8255/3, 8211/3, 8260/3, 8263/3), signet ring cell carcinoma (8490/3) and other uncommon pathological classification. Patients were excluded if they had multiple primary malignant neoplasms, incomplete TNM staging, with distant metastasis (M1), no evaluation on LNs, died within 30 days after surgery or information on CSS and survival months unavailable.

Age, sex, year of diagnosis, race, grade, T stage, total number of LN examined, number of positive LNs and survival time was assessed. TNM classification was restaged according to the criteria described in the AJCC Cancer Staging Manual (7th edition, 2010).

### Statistical analysis

The NLNs cutoff points were determined using the X-tile program, which identified the cutoff with the minimum P values from log-rank χ2 statistics for the categorical NLNs in terms of survival. Association of ypN stage with clinicopathological parameters was analyzed by chi-square (χ2) test. Survival curves were generated using Kaplan-Meier estimates, and the differences were analyzed by log-rank test. Cox regression models were built for analysis of risk factors for survival outcomes. Nomogram on CSS was established according to all significant factors. Statistical analyses were performed using the statistical software package SPSS for Windows, version 19.0 (SPSS Inc, Chicago, IL, USA). Results were considered statistically significant when a two-sided p values of less than 0.05.
